# Special Issue “Molecular Mechanisms and Therapeutic Targeting of Inflammatory Complications”

**DOI:** 10.3390/ijms27209139

**Published:** 2026-10-15

**Authors:** Kota V. Ramana

**Affiliations:** Department of Biomedical Sciences, Noorda College of Osteopathic Medicine, Provo, UT 84606, USA; karamana@noordacom.org

During infections and injuries, the body generally reacts by releasing inflammatory cytokines to fight against invading pathogens. This natural immune response is called inflammation [1]. Although inflammation protects the body from infection, tissue injury, and other harmful stimuli, uncontrolled inflammation and the production of inflammatory cytokines and chemokines through autocrine and paracrine mechanisms cause tissue damage and dysfunction, leading to inflammatory complications [2,3]. Thus, persistent activation of immune and oxidative responses due to impaired inflammatory resolution leads to a wide range of human inflammatory disorders. Generally, during infections, recognition of pathogen-associated molecular patterns (PAMPs) and damage-associated molecular patterns (DAMPs) by pattern-recognition receptors (PRRs), such as Toll-like receptors, NOD-like receptors, RIG-I-like receptors, and C-type lectin receptors [4,5,6], initiates an inflammatory response. Further, activation of these receptors also triggers the nuclear factor kappa B (NF-κB) signalosome and NLRP3 inflammasome pathways, which are important for regulating host defense [7,8]. Thus, immune cell activation and immune responses during pathological conditions eliminate pathogens and damaged cells and restore homeostasis [9,10]. Inflammation is therefore indispensable for effective host defense and recovery from injury, rather than being essentially pathological. However, if the initiating stimulus persists, a protective response may become chronic [9,10], resulting in tissue damage, fibrosis, metabolic dysfunction, and eventually organ dysfunction.

It is well known that oxidative stress and inflammation are closely linked processes. Generally, reactive oxygen species (ROS) can play important physiological roles in redox signaling and in antimicrobial defense. However, excessive ROS production can damage proteins, lipids, and nucleic acids [11,12]. During infections, neutrophils and macrophages generate significant amounts of ROS and activate redox-sensitive signaling pathways. These redox pathways further regulate the inflammatory response, linking oxidative stress and inflammation in a reciprocal relationship [13].

Recent studies also suggest that metabolic disturbances could regulate the relationship between inflammation and immune cell activation. Metabolic pathways function not only as sources of cellular energy but also as key regulators of immune cell differentiation, activation, and effector function [14]. Therefore, alterations in glycolysis, oxidative phosphorylation, fatty acid metabolism, and mitochondrial function can modify cellular metabolism and cause redox imbalance [14,15]. Thus, immune dysregulation, metabolic imbalance, and oxidative stress are interconnected processes that contribute to chronic inflammation and mediate disease progression. Consequently, current strategies should target specific molecular pathways that sustain pathological inflammation and correct dysregulated metabolic and redox signaling.

These mechanisms are clinically significant because persistent or dysregulated inflammation contributes to the pathogenesis and complications of numerous acute and chronic diseases. Chronic inflammation is central to cardiovascular and metabolic diseases, autoimmune disorders, respiratory diseases, and neurological and other chronic inflammatory conditions. Recent advances in proteomics, metabolomics, lipidomics, and epigenetics technologies are uncovering novel biomarkers within inflammatory pathways and immune cell populations. These developments help us understand and treat inflammatory complications by targeting specific receptors and signaling pathways that regulate inflammation and restore metabolic and redox balance.

This Special Issue, titled “Molecular Mechanisms and Therapeutic Targeting of Inflammatory Complications”, presents fifteen contributions, including eight research articles and seven review articles, that illustrate how inflammation, metabolism, and oxidative stress lead to various inflammatory complications. Collectively, these studies address important complications across a range of human inflammatory disorders. Through integrating mechanistic studies with biomarker discovery and emerging clinical strategies, this Special Issue offers a comprehensive perspective on the molecular basis of pathological inflammation. It points out potential ways to predict, prevent, and target inflammation-associated diseases.

An interesting research study by Kirilova et al. [Contribution 1] evaluated whether preoperative inflammatory and hypoxia-related biomarkers could predict postoperative inflammatory complications, specifically in patients undergoing cardiac surgery. They found that patients who subsequently developed complications had higher eosinophil counts, increased HIF1A expression, and a higher HIF1A/EPAS1 ratio, whereas neutrophil counts and the neutrophil-to-lymphocyte ratio were lower. These data suggest the value of integrating inflammatory and hypoxia-responsive molecular markers into preoperative risk assessment and indicate that inflammatory complications may be identifiable at the molecular level before clinical manifestation. Similarly, Yarbrough et al. [Contribution 2] demonstrate the close relationship between inflammation, metabolism, and aging. Using young and old mice models of periodontitis, the authors examined the effects of the CD38-specific inhibitor 78c. The results show that the treatment increased NAD+ levels in the liver and spleen of both age groups and increased maxillary NAD+ levels in older animals. Importantly, CD38 inhibition also reduced alveolar bone loss in both young and old mice. These data link age-related changes in NAD+ metabolism to inflammatory tissue destruction and support CD38 inhibition as a possible strategy to limit periodontal disease.

Another research study by Andreeva et al. [Contribution 3] investigated the clinical and inflammatory characteristics of SARS-CoV-2 infection in people living with HIV (PLWH). In this pilot study, PLWH with COVID-19 demonstrated a greater occurrence of severe disease than patients with COVID-19 alone, and four fatal cases occurred in the PLWH/COVID-19 group. The authors also showed that circulating concentrations of several pro-inflammatory cytokines, including IL-5, IL-6, IL-9, and IL-15, were elevated in the coinfected group. Although the preliminary nature of the study warrants careful interpretation, these results indicate that altered cytokine responses may contribute to the increased disease severity observed in PLWH with COVID-19.

A fascinating study by Bonuccelli et al. [Contribution 4] examined the relationship between inflammatory signaling and systemic metabolic homeostasis in inflammatory bowel disease (IBD)-associated anemia. IL-6 is an important inflammatory mediator that promotes expression of HAMP, the gene encoding hepcidin, thereby contributing to disturbed iron metabolism during chronic inflammation. In this study, the authors tested the effects of the enterolignans enterodiol (END) and enterolactone (ENL) in IL-6-treated Caco-2 cells. The results indicate that END and ENL reduced IL-6-induced HAMP expression and partially restored FTH1 and ACO1 gene expression. This study thus suggests that enterolignans could modulate the interface between inflammatory signaling and iron homeostasis in IBD-associated anemia. On the other hand, Park et al. [Contribution 5] also showed that naturally derived compounds can simultaneously modulate inflammation and tissue dysfunction. The authors evaluated an ethanol extract of Carica papaya peel (EECP), which contains several bioactive compounds with antioxidant and anti-inflammatory properties, in a mouse model of contact dermatitis. EECP improved visible skin lesions and measures of skin barrier function while reducing epidermal hyperplasia, spongiotic changes, and inflammatory-cell infiltration. EECP also suppressed TNF-α, IFN-γ, IL-6, and MCP-1 production, restored filaggrin expression, and inhibited ERK phosphorylation and ICAM-1 expression. These data show the significance of C. papaya peel-derived compounds for inflammatory skin disorders.

Further, an intriguing study by Min et al. [Contribution 6] investigated the active promotion of inflammatory resolution in radiation-induced lung inflammation (RILI), a clinically important complication of thoracic radiotherapy that can progress from acute inflammation to pulmonary fibrosis. Specifically, the authors evaluated the role of CYNC-2, a newly synthesized analog of the specialized pro-resolving mediator lipoxin A4. They have shown that CYNC-2 activates AMPK, inhibits mTOR signaling, and suppresses NLRP3 inflammasome activation. It also reduced the production of inflammatory mediators such as IL-1β, IL-6, and TGF-β1. This study thus suggests that stable analogs of endogenous pro-resolving mediators may yield a promising strategy to limit inflammatory complications of radiotherapy. Another research study by Kagelmacher et al. [Contribution 7] investigated the effects of dendritic polyglycerol sulfate (dPGS) on signaling mediated by high-mobility group box 1 (HMGB1), a damage-associated molecular pattern released from stressed or damaged cells. The data showed that in macrophages and human microglia, dPGS disrupted HMGB1/RAGE interactions and reduced NF-κB activation. Further, dPGs also decreased transcription of TNF-α, IL-6, MCP-1, COX-2, and iNOS. This study provides mechanistic evidence that directly interfering with danger signal/receptor interactions can attenuate downstream inflammatory signaling and may offer a strategy for controlling HMGB1-associated inflammatory responses. Additionally, Tourountzis et al. [Contribution 8] examined protein-bound uremic toxins (PBUTs), lymphocyte immunosenescence, and clinical outcomes in patients receiving hemodialysis. During two years of follow-up, increased free indoxyl sulfate and higher total and free hippuric acid concentrations were associated with mortality, while higher concentrations of CMPF were observed in patients who experienced cardiovascular events. These findings indicate the potential clinical importance of metabolic toxins as contributors to the inflammatory abnormalities accompanying advanced kidney disease.

An interesting review article by Zhao et al. [Contribution 9] discussed the significance of exosomes in gouty arthritis. Specifically, the authors also discussed the translational potential of engineered exosomes in targeted drug delivery, immunomodulation, and disease monitoring. This review suggests extracellular vesicle biology as an emerging interface among inflammatory mechanisms, biomarkers, and precision therapeutics. Similarly, another review article by Gerasimova et al. [Contribution 10] analyzed the functions of tripartite motif (TRIM) proteins in neuroinflammation and peripheral inflammatory diseases. TRIM proteins participate in innate immune signaling, protein turnover, cellular stress responses, and inflammatory regulation. Their functions appear to differ by cell type. In microglia, they are frequently associated with intracellular inflammatory signaling, whereas neuronal TRIM proteins are more commonly linked to cell survival and oxidative stress. The authors also emphasized that individual TRIM proteins may exert pro- or anti-inflammatory effects depending on biological need, and in some cases their reported functions remain contradictory. Knowing these cell- and disease-specific effects will therefore be essential before individual TRIM proteins can be effectively targeted therapeutically. A scoping and evidence-mapping review article by Santos et al. [Contribution 11] evaluated saliva as a non-invasive source of IBD biomarkers. The authors have included 12 studies across eight molecular and microbial biomarker categories. The authors demonstrated the most consistent potential of cytokines, microRNAs, calprotectin, and microbiome alterations. They also showed that changes such as increased Prevotella abundance and elevated PSMA7 levels could also alter intestinal barrier integrity and immune activity. This review indicates that standardized approaches and further clinical validation are still needed before routine implementation.

The pathophysiology of cardiovascular disease also involves a balance between inflammation initiation and resolution, which further influences chronic disease progression. A review article by Chistyakov et al. [Contribution 12] discussed the significance of bioactive lipid mediators, oxylipins, in atherosclerosis. The oxylipins generally regulate vascular function, leukocyte recruitment, and plaque stability. The authors suggest that the balance between pro-inflammatory oxylipins and specialized pro-resolving mediators is particularly important in atherosclerotic progression. Thus, advances in lipidomic profiling increasingly enable the identification of oxylipin signatures associated with coronary artery disease. Another interesting review article by Zolotarenko et al. [Contribution 13] discussed the significance of epigenetic regulation during SARS-CoV-2 infection. The authors describe how viral infection can alter host DNA methylation, microRNA profiles, and other epigenetic regulatory mechanisms. They also indicate how epigenetic regulation controls antiviral responses, disease severity, and potentially the persistent manifestations of long COVID. The authors indicate that candidate epigenetic biomarkers, including methylation signatures involving AIM2, HLA-C, and PARP9, as well as dysregulated microRNAs, may offer additional ways to understand disease heterogeneity. Thus, this review article discusses how acute infectious inflammatory stimulus may leave persistent molecular changes that influence immune function well beyond the initial infection.

Another article by Zafar et al. [Contribution 14] discussed the therapeutic potential of curcumin, a polyphenolic compound extracted from Curcuma longa, across a broad range of inflammatory diseases. Specifically, the authors discussed the significance of curcumin use in osteoarthritis, asthma, chronic obstructive pulmonary disease, atherosclerosis, irritable bowel syndrome, sepsis, and atopic dermatitis. The authors note that although experimental and clinical observations are encouraging, rigorous, adequately powered studies are needed to establish efficacy and guide clinical application. Thus, this review highlights the therapeutic promise of the multi-target natural compound curcumin in managing inflammatory diseases. Finally, a comprehensive review article by Ortega-Zapero et al. [Contribution 15] discussed the significance of neutrophils and neutrophil extracellular traps (NETs) in IBD. Neutrophils contribute to intestinal inflammation by producing ROS, inflammatory cytokines, and NETs. They discussed how controlled NET formation helps antimicrobial defense and tissue repair, and how excessive NET formation can damage the epithelial barrier and increase thrombotic risk. In this article, the authors also discussed therapeutic methods including PAD4 inhibitors, modulation of NADPH oxidase and ROS pathways, and DNase I-mediated NET degradation.

Taken together, the contributions included in this Special Issue demonstrate that inflammatory complications emerge from a highly interconnected network mainly involving immune activation, metabolic imbalance, and oxidative stress. These studies indicate the importance of suppressing inflammatory responses, as well as specifically modulating molecular pathways, in controlling various inflammatory complications. Effectively managing inflammatory complications will require integrating mechanistic biology with biomarker discovery, systems-level analysis, and carefully validated clinical interventions. Thus, the studies reported in this Special Issue highlight the molecular mechanisms, biomarkers, and therapeutic approaches that could help us improve the prevention and management of inflammation-driven human diseases.
